# Dietary RNA from Torula Yeast Prevents Capillary Regression in Atrophied Skeletal Muscle in Rats

**DOI:** 10.3390/life14121616

**Published:** 2024-12-06

**Authors:** Hao Lin, Jihao Xing, Xiaoqi Ma, Ryosuke Nakanishi, Hiroyo Kondo, Mica Fujita, Keita Sutoh, Noriaki Maeshige, Hidemi Fujino

**Affiliations:** 1 Department of Rehabilitation Science, Kobe University Graduate School of Health Sciences, 7-10-2, Tomogaoka, Suma-ku, Kobe 654-0142, Japan; linhao_china1995@yahoo.co.jp (H.L.); xingjihaozibo@163.com (J.X.); mxq213k212k@gmail.com (X.M.); rnakanishi@kobe-kiu.ac.jp (R.N.); kondohiroyo2@gmail.com (H.K.); nmaeshige@pearl.kobe-u.ac.jp (N.M.); 2 Fordays Co., Ltd., Koami-cho, Nihonbashi, Chuo-ku, Tokyo 103-0016, Japan; m.fujita@fordays.jp (M.F.); k.sutoh@fordays.jp (K.S.); 3 Tokyo University of Agriculture and Technology Center for Advanced Industry-Academia Collaborative Research, Tokyo University of Agriculture and Technology, 2-24-16 Naka-cho, Koganei, Tokyo 184-8588, Japan

**Keywords:** RNA, angiogenic factors, oxidant stress, capillary regression

## Abstract

Chronic neuromuscular inactivity induces capillary regression within skeletal muscle. The objective of this study was to investigate the potential effects of dietary nucleic acids in counteracting the capillary reduction linked to chronic neuromuscular inactivity in the soleus muscle. The study utilized four distinct groups of female Wistar rats: a control group (CON), a hindlimb-unloading group (HU), an HU group supplemented with DNA (HU + DNA), and an HU group supplemented with RNA (HU + RNA). For a duration of two weeks, rats in the HU + DNA and HU + RNA groups were administered 1500 mg/kg of DNA or RNA orally on a daily basis. Two weeks of hindlimb unloading was concomitant with a reduction in the absolute weight of the soleus muscle and the capillary-to-fiber (C/F) ratio. This was associated with changes due to disuse, including increased accumulation of reactive oxygen species (ROS) and reduced levels of superoxide dismutase (SOD-2), along with elevated levels of thrombospondin-1 (TSP-1), an anti-angiogenic factor. Administering DNA at a medium concentration in the diet did not effectively prevent the reduction in the ratio between capillaries and fibers. In contrast, the equivalent concentration of RNA successfully averted the regression of capillaries during the unloading phase. Additionally, reactive oxygen species (ROS), superoxide dismutase-2 (SOD-2), and thrombospondin-1 (TSP-1) protein were kept at the same levels as in the control. The aforementioned findings reveal that RNA is more effective than DNA in preventing capillary regression triggered by muscle atrophy.

## 1. Introduction

Capillaries, as mediators of gas, fluid, and nutrient exchange, are indispensable for the optimal physiological functioning of the skeletal muscle system. Additionally, the connection between capillaries and myofibers plays an essential role in the functioning of the muscular system. Indeed, it serves as the point at which waste products, heat from metabolism, nutrients, and oxygen are exchanged between the blood and the muscle fibers [1]. Under various conditions, capillary regression and growth can be modulated in skeletal muscle. Although exercise promotes the development of the network of capillaries in healthy individuals [2], physical deterioration and diabetes lead to a decline in the capillary network in skeletal muscle [3,4].

The rodent model of hindlimb unloading (HU) is extensively used to examine conditions such as hypokinesia and hyperdynamic states [5], enabling investigations into diverse aspects of muscular loading [6,7]. HU significantly reduced the quantity of capillaries in the soleus muscle in comparison to the plantaris muscle [4], which mostly contains oxidative slow-twitch fibers and shows significant vulnerability to unloading conditions. Additionally, numerous scholars have documented that HU induces a pronounced reduction in both capillary dimensions and density within the soleus muscle under unloading conditions [8,9]. These physiological changes are predominantly attributed to a dysregulation in the balance of reactive oxygen species (ROS) production and antioxidant defense mechanisms, ultimately leading to oxidative stress in muscles subjected to disuse [10,11].

Extensive research demonstrates that oxidative stress plays a pivotal role in mediating the upregulation of negative angiogenic regulators, with thrombospondin-1 (TSP-1) being the most prominent, particularly in skeletal muscles undergoing disuse [12]. TSP-1 functions as an anti-angiogenic regulator and acts as a matrix glycoprotein synthesized by multiple cell types, including endothelial cells. Through its CD36 receptor, TSP-1 can bind and subsequently inhibit the activation of the VEGF-A signaling cascade. Its role is intricately linked to the process of capillary regression [13]. Additionally, the administration of the TSP-1 has been shown to reduce capillarity in skeletal muscles in previous study [14]. The regression of capillaries also observed in the rat soleus muscle under unloading conditions is largely mediated by the influence of TSP-1 [4].

In previous studies, it was ascertained that the administration of an antioxidant via dietary supplementation demonstrated efficacy in preventing the decline of capillaries in dormant muscles. This achievement was attributed to the rectification of the disparity between factors that facilitate and impede the growth of blood vessels. Nucleoproteins, comprising DNA extracted from salmon milt, exhibit significant antioxidant properties and have been shown to attenuate stress-induced neuroinflammation in the brains of aged mice [15]. Our previous research suggests that high concentration of DNA can prevent the mitochondrial dysfunction and capillary regression associated with hindlimb unloading [16]. Similarly, monoribonucleotides from yeast extract have been found to enhance the antioxidant response in Nile Tilapia [17]. Both DNA and RNA share a fundamental structure consisting of a nitrogenous base, a five-carbon sugar, and one or more phosphate groups [18]. However, while the antioxidant effects of RNA are well-documented, their impact on capillary regression remains unclear. The present study seeks to examine the impact of RNA on capillary regression in comparison to DNA. We hypothesize that administering DNA or RNA to hindlimb-unloaded rats will reduce oxidative stress in the inactive muscle, thereby protecting muscles from capillary regression and atrophy.

## 2. Materials and Methods

### 2.1. Experimental Group

Twenty-four 12-week-old female Wistar rats (Japan SLC, Hamamatsu, Japan; 191.6 ± 10.5 g body weight) were employed in the present study. The rats were randomly assigned to four different groups: (1) Control (CON); (2) Hindlimb-unloading (HU); (3) Hindlimb-unloading with DNA administration (HU + DNA); (4) Hindlimb-unloading with RNA administration (HU + RNA). The rats in the HU + DNA and HU + RNA groups were orally administered DNA or RNA (1500 mg/kg/day with a 10 h time span between two daily doses, Nissan Chemical Industries, Tokyo, Japan) solubilized in distilled water using a catheter, twice daily. Animals in the other experimental groups were orally administered distilled water. After a one-week period of administration for adaptation, the rats in the HU, HU + DNA, and HU + RNA groups were unloaded for 2 weeks. The animals were kept in an isolated, climate-controlled room at 22 ± 2 °C, following a 12–12 h light–dark cycle, and were provided with food and water ad libitum.

### 2.2. Hindlimb Unloading

The hindlimb unloading procedure was conducted based on the method described by [19], with adjustments. Briefly, the rats were suspended by their tails with adhesive tape and a kite string, ensuring that their hindlimbs remained elevated, without contact with the cage floor or walls. The forelimbs were maintained in contact with the floor of the cage throughout the procedure.

### 2.3. Sample Preparation

Approximately 24 h following the final oral administration, all rats were deeply anesthetized with 4% isoflurane via inhalation and humanely euthanized through intraperitoneal injection of sodium pentobarbital (50 mg/kg). The soleus muscles were dissected, weighed, rapidly stored in a dry ice–acetone bath, and then transferred to −80 °C for subsequent analysis.

The DNA and RNA samples were supplied by Fordays Co., Ltd. (Tokyo, Japan) DNA was extracted from salmon milt and hydrolyzed by protease for water solubility. DNA concentration in the DNA sample was about 70%. RNA was extracted from torula yeast (*Cyberlindnera jadinii*). RNA concentration in the RNA sample was about 70%. The RNA and DNA concentrations in each material were analyzed by HPLC after nuclease treatment for digestion of the nucleic acids. The concentrations of mono-ribonucleotides and mono-deoxyribonucleotides (free nucleotides) were estimated using non-nuclease-treated samples. The ribonucleotide and deoxyribonucleotide concentrations obtained from RNA or DNA (after nuclease treatment) are showed as RNA and DNA concentrations (Table 1).

### 2.4. Histological Analysis

Ten-micrometer-thick soleus muscle sections were cut from the mid-portion of the right-side muscle in a cryostat at −25 °C using a CM-1510S cryostat (Leica Microsystems, Mannheim, Germany) and mounted on glass slides. To visualize capillaries, the transverse sections were stained for alkaline phosphatase (AP), incubated with 5-bromo-4-chloro-3-indolyl phosphate/nitro blue tetrazolium for 45 min at 37 °C, and fixed using 4% paraformaldehyde. The capillary-to-fiber (C/F) ratio was quantified by counting capillaries and myofibers in AP-stained cryosections, utilizing high-resolution microscopic imaging. Microscopic analysis was used to determine the capillary-to-fiber (C/F) ratio by quantifying the capillaries and muscle fibers present in each cryosection. Over 100 fibers per muscle sample from all animals were analyzed to guarantee accuracy. The data collection was performed using the ImageJ software (1.53a) rovided by the NIH (NIH, Bethesda, MD, USA).

As previously reported by [20], the sections were stained with succinate dehydrogenase (SDH) to assess the oxidative capacity of skeletal muscle. The sections were incubated at 37 °C for 45 min in 0.1 M phosphate buffer (pH 7.6) with 0.9 mM NaN_3_, 0.9 mM 1-methoxy phenazine methylsulfate, 1.5 mM nitroblue tetrazolium, 5.6 mM EDTA-disodium, and 48 mM succinate disodium. SDH activity for each group was assessed via densitometric analysis. As previously reported by [21], myofibrillar ATPase staining was applied to other sections, preincubated at pH 4.2, to classify the muscle fiber types. These sections were subsequently analyzed to determine the fiber cross-sectional area (FCSA) and the proportion of slow-twitch fibers. The ratio of dark-stained fibers to the total number of fibers was calculated to assess slow-twitch fiber composition. The cross-sectional area (CSA) of individual muscle fibers was measured using ImageJ software.

To determine the intracellular ROS levels, the redox-sensitive probe dihydroethidium (DHE) (Wako Pure Chemicals, Osaka, Japan) was used, which is oxidized to ethidium by superoxide. DHE permeates cells and, in the presence of O_2_, is converted to ethidium bromide, which binds to nuclear DNA and emits red fluorescence proportional to the O_2_ levels (excitation at 488 nm) [22,23]. The soleus muscles were cut into 10 μm sections. Sections from all groups were incubated with 5 μM DHE (dissolved in dimethylsulphoxide) for 30 min at 37 °C in the dark, followed by fixation in 2% phosphate-buffered paraformaldehyde for 15 min. Fluorescence microscopy (BX51; Olympus, Tokyo, Japan) was used for visualization, and grey scale analysis was employed to quantify the fluorescence area fraction using two images per section, with one section analyzed per muscle. Although this method for DHE staining has been widely used in skeletal muscle research [22,23,24], some limitations exist. Freezing and cutting sections while exposing them to DHE could artificially trigger ROS release. However, this effect was likely minimized, as all samples were handled similarly.

### 2.5. SDS Polyacrylamide Gel Electrophoresis and Western Blot Analysis

Muscle tissue samples were homogenized on ice using a buffer composed of 50 mmol/L Tris-HCl (pH 8.0), 120 mmol/L NaCl, 1% NP-40, 20 mmol/L NaF, 1 mmol/L EDTA, 1 mmol/L EGTA, 15 mmol/L sodium pyrophosphate, 30 mmol/L β-glycerophosphate, 2 mmol/L Na_2_VO_4_, and 1% protease inhibitor cocktail. After homogenization, centrifugation of the samples was performed at 1700× *g* for 10 min at 4 °C, and the supernatants were obtained. Protein concentration in the supernatants was then determined as described previously. The supernatants were subsequently mixed with a sample loading buffer (50 mmol/L Tris–HCl, pH 6.8, 2% SDS, 10% glycerol, 5% 2-mercaptoethanol, and 0.005% bromophenol blue) and heated at 80 °C for 10 min.

Proteins (30 µg per lane) were separated using SDS polyacrylamide gel electrophoresis (SDS-PAGE) and then transferred onto PVDF membranes for further analysis. To block non-specific binding, the membranes were treated with 5% skimmed milk in PBST (phosphate-buffered saline containing Tween-20) for 1 h. The membranes, following blocking, were incubated with primary antibodies, including anti-VEGF (1:200 in PBST, sc-7269; Santa Cruz Biochemistry, Dallas, TX, USA), anti-TSP-1 (1:100 in PBST, sc-59887; Santa Cruz Biochemistry), anti-SOD-1 (1:200 in PBST, sc-8637; Santa Cruz Biotechnology), anti-SOD-2 (1:200 in PBST, #13141; Cell Signaling Technology, Tokyo, Japan), and anti-GAPDH (1:1000 in PBST, sc-32233; Santa Cruz Biochemistry). Detection of the primary antibodies was performed with horseradish peroxidase-linked anti-rabbit or anti-mouse IgG (GE Healthcare, Waukesha, WI, USA), followed by visualization with the Ez West Lumi chemiluminescent reagent (ATTO, Tokyo, Japan) and imaging on the LAS-1000 system (Fujifilm, Tokyo, Japan).

### 2.6. Determination of SOD-like Activity Induced by Dietary DNA and RNA

Through application of the SOD assay kit WST, sourced from Dojindo Molecular Technologies Inc. (Gaithersburg, MD, USA), SOD-like activity in the DNA and RNA supplements and in homogenized muscle tissue were measured as per the protocol provided by the manufacturer. Superoxide radical scavenging in the xanthine/xanthine oxidase system was evaluated by measuring the reduction of WST-1 (2-(4-iodophenyl)-3-(4-nitrophenyl)-5-(2,4-disulphophenyl)-2H-tetrazolium, monosodium salt) using spectrophotometry. The EC50, defined as the concentration needed to inhibit WST-1 formazan production by 50%, was averaged across four experiments. One SOD unit was defined as the enzyme quantity that resulted in 50% inhibition of WST-1.

### 2.7. Statistical Analysis

Data are presented as mean ± SEM. Intergroup differences were analyzed via one-way ANOVA, with Tukey’s post hoc test for pairwise comparisons, setting statistical significance at *p* < 0.05.

## 3. Results

### 3.1. Body Mass, Soleus Muscle Mass, FCSA (Fiber Cross-Sectional Area), and Composition of Slow-Twitch Fibers

**Figure 1:**
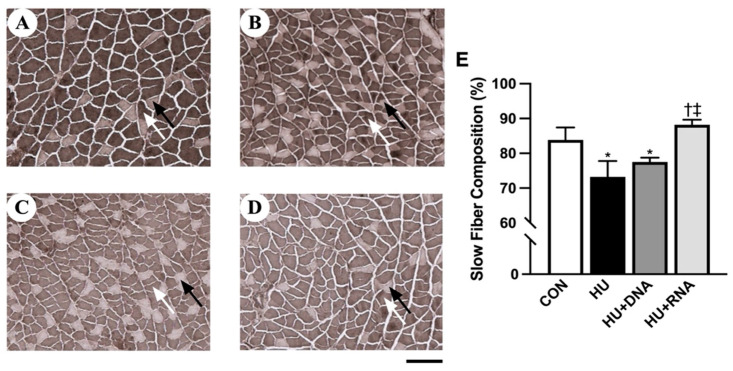
Transverse sections were stained for adenosine triphosphate (ATPase) (**A**–**D**, pH: 4.2) to determine the slow fiber composition (**E**) of the soleus muscles. CON (**A**), HU (**B**), HU +DNA (**C**), HU + RNA (**D**). Bar = 50 μm. Values are means ± SEM (n = 6). CON, control; HU, hindlimb-unloaded; HU + DNA, hindlimb-unloaded plus DNA; HU + RNA, hindlimb-unloaded plus RNA. Light staining (Black arrow): type II; Dark staining (White arrow): type I. The symbols *, †, and ‡ indicate significant differences with respect to the CON, HU, and HU + DNA groups, respectively, at *p* < 0.05. The RNA treatment attenuated the HU-induced transformation from slow to fast muscle type.

A 14-day hindlimb unloading period led to notable decreases in mean body mass, absolute soleus muscle mass (*p* < 0.05 vs. CON group), fiber cross-sectional area (*p* < 0.05 vs. CON group) (Table 2), and proportion of slow fibers (*p* < 0.05 vs. CON group) (Figure 1E). Neither the DNA nor the RNA intervention effectively attenuated the loss of soleus muscle mass (*p* = 0.9679 and *p* = 0.9237 vs. HU group, respectively) and FCSA (*p* = 0.2183 and *p* = 0.1511 vs. HU group, respectively). Although the treatment with DNA did not prevent the reduction in slow fiber composition (*p* = 0.1005 vs. HU group), the treatment with RNA prevented the reduction in slow fiber composition (*p* < 0.05 vs. HU group).

### 3.2. Capillary Count

**Figure 2:**
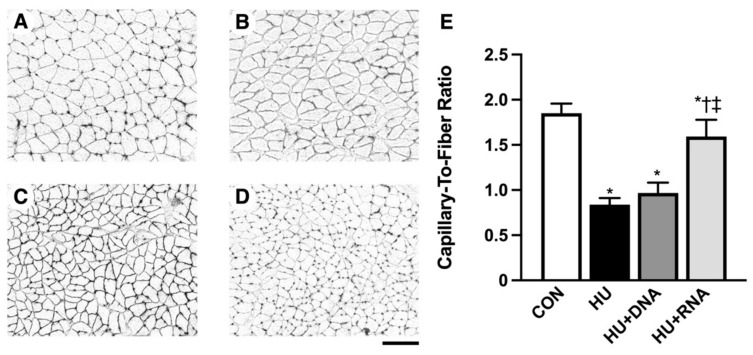
Transverse sections stained for alkaline phosphatase (**A**–**D**) and C/F ratio (**E**) of the soleus muscles. CON (**A**), HU (**B**), HU + DNA (**C**), HU + RNA (**D**). Capillaries are visualized as black dots. Bar = 50 μm. Values are means ± SEM (n = 6). CON, control; HU, hindlimb-unloaded; HU + DNA, hindlimb-unloaded plus DNA; HU + RNA, hindlimb-unloaded plus RNA. The symbols *, †, and ‡ indicate significant differences with respect to the CON, HU, and HU + DNA groups, respectively, at *p* < 0.05. HU harmed the capillary network, while the RNA treatment prevented capillary regression in the skeletal muscle.

Figure 2A–E illustrates a typical image depicting the dyed capillary within the soleus muscles. The capillary-to-fiber (C/F) ratio was significantly reduced after 14 days of HU (*p* < 0.05 vs. CON group). The reduction in the C/F ratio was avoided with the RNA treatment (HU + RNA) (*p* < 0.05 vs. HU group), while the DNA treatment (HU + DNA) failed to prevent this decrease (*p* = 0.3209 vs. HU group) (Figure 2E).

### 3.3. Pro- and Antiangiogenic Factors

**Figure 3:**
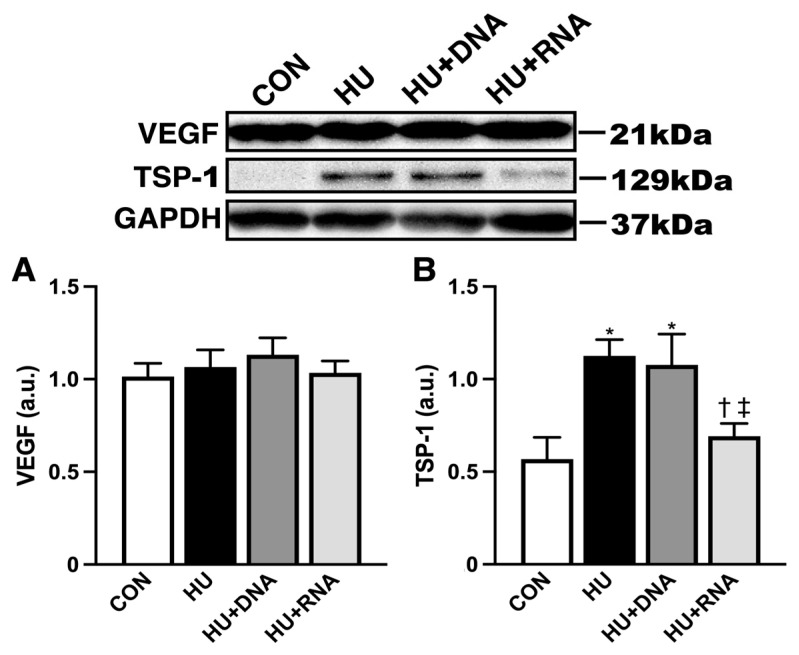
RNA treatment inhibits the antiangiogenic factor TSP-1 in rats with hindlimb-unloading-induced atrophy. (**A**) Western blot result of VEGF expression and comparison of VEGF protein expression between the groups. (**B**) Western blot result of TSP-1 expression and comparison of TSP-1 protein expression between the group. Values were calculated as fold changes relative to the control group. Values are expressed as mean ± SEM (n = 6). The symbols *, †, and ‡ indicate significant differences with respect to the CON, HU, and HU + DNA groups, respectively, at *p* < 0.05.

SDS-PAGE and Western blot techniques were employed to analyze the protein contents of VEGF and TSP-1. No notable variations (HU, HU + DNA, HU + RNA groups) were observed in the VEGF protein levels compared to the CON group (*p* = 0.9672, *p* = 0.7265, and *p* = 0.9978, respectively) (Figure 3A). Simultaneously, the expression level of the TSP-1 protein, an antiangiogenic factor, exhibited a significant increase following a 14-day period of HU (*p* < 0.05 vs. CON group) (Figure 3B). DNA supplementation (HU + DNA) was ineffective in mitigating the HU-induced alterations (*p* = 0.9721 vs. HU group), whereas the RNA treatment (HU + RNA) demonstrated a significant ability to suppress these changes (*p* < 0.05 vs. HU group).

### 3.4. Oxidative Enzyme Activity

**Figure 4:**
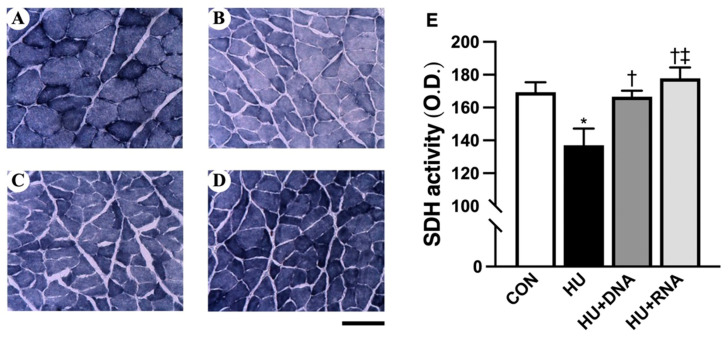
Transverse sections stained for succinate dehydrogenase (SDH) (**A**–**D**) and SDH activity (**E**) in the soleus muscles. CON (**A**), HU (**B**), HU + DNA (**C**), HU + RNA (**D**). Bar = 100 μm. Values are means ± SEM (n = 6). CON, control; HU, hindlimb-unloaded; HU + DNA, hindlimb-unloaded plus DNA; HU + RNA, hindlimb-unloaded plus RNA. The symbols *, †, and ‡ indicate significant differences with respect to the CON, HU, and HU + DNA groups, respectively, at *p* < 0.05. The activity of mitochondrial oxidase was reduced by HU and improved by the RNA treatment.

The activity of succinate dehydrogenase (SDH) was measured via SDH staining. SDH activity showed a marked decrease after 14 days of HU (*p* < 0.05 vs. CON group) (Figure 4E). The enzyme activity in muscle exhibited significant increases in both the HU + DNA (*p* < 0.05) and the HU + RNA groups (*p* < 0.05) in comparison with the HU group.

### 3.5. Effect of DNA and RNA on SOD-like Activity

**Figure 5:**
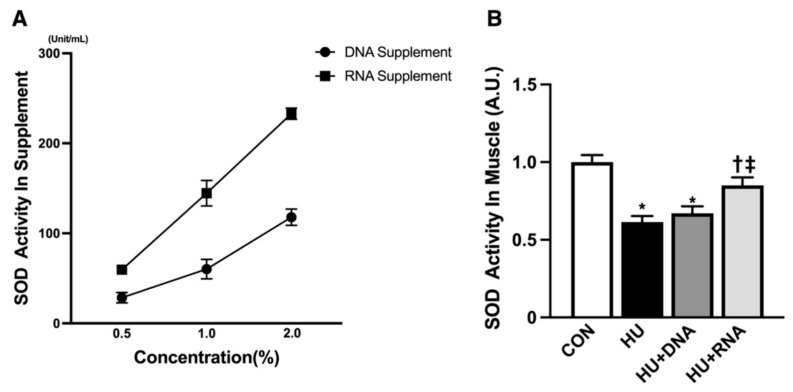
Effect of RNA treatment on superoxide radical scavenging activity compared to DNA treatment. (**A**) RNA supplements are more protective against oxidative stress than DNA supplements in various concentrations. (0.5%, 1%, and 2%) (**B**) RNA treatment attenuates hindlimb-unloading-induced production of superoxide radicals in soleus muscle. Values are means ± SEM (n = 6). CON, control; HU, hindlimb-unloaded; HU + DNA, hindlimb-unloaded plus DNA; HU + RNA, hindlimb-unloaded plus RNA. The symbols *, †, and ‡ indicate significant differences with respect to the CON, HU, and HU + DNA groups, respectively, at *p* < 0.05.

To assess the antioxidant properties of DNA and RNA dietary supplementation, DNA (0.5%, 1%, 2%) and RNA (0.5%, 1%, 2%) were prepared. Their superoxide radical scavenging activity was ranked in the following sequence: RNA 2% > RNA 1% > DNA 2% > RNA 0.5% = DNA 1% > DNA 0.5% (Figure 5A).

And the antioxidant effect of DNA and RNA on muscle was also evaluated. The level of SOD activity was significantly reduced by 14-day HU (*p* < 0.05 vs. CON group). Despite the ineffectiveness of DNA supplementation (HU + DNA) in preventing the HU-induced changes (*p* = 0.8131 vs. HU group), the RNA treatment (HU + RNA) demonstrated a significant inhibitory effect (*p* < 0.05 vs. HU group) (Figure 5B).

### 3.6. Oxidative Stress Level

**Figure 6:**
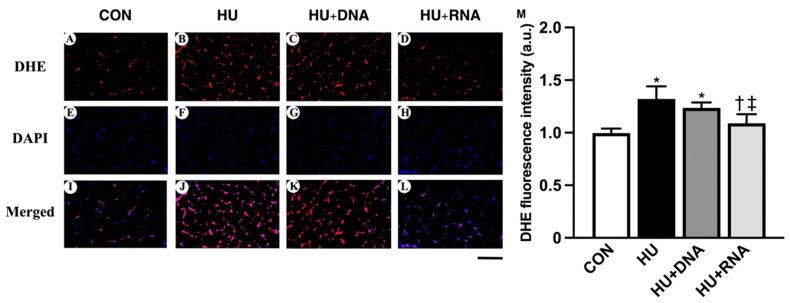
Transverse sections from each group were stained with the fluorescent probe dihydroethidium (DHE) that detects oxidative stress. Representative images of dihydroethidium (**A**–**D**) and DAPI staining (**E**–**H**) and merged images (**I**–**L**) of the soleus muscle in each group. Mean levels of DHE fluorescence (**M**) in the soleus muscle of each group. Scale bar: 100 μm. Values are means ± SEM (n = 6). CON, control; HU, hindlimb-unloaded; HU + DNA, hindlimb-unloaded plus DNA; HU + RNA, hindlimb-unloaded plus RNA. The symbols *, †, and ‡ indicate significant differences with respect to the CON, HU, and HU + DNA groups, respectively, at *p* < 0.05.

The generation of intracellular ROS was evaluated using nuclear DHE fluorescence. Typical images depicting the soleus muscle stained with DHE for each group are presented in Figure 6A–D. The DHE fluorescence levels in the HU group were significantly elevated compared to those in the CON group (*p* < 0.05). However, the RNA treatment (HU + RNA) prevented this elevation (*p* < 0.05 vs. HU group). In contrast, the DNA treatment (HU + DNA) did not restrain the upward trend (*p* = 0.2979 vs. HU group) (Figure 6M).

**Figure 7:**
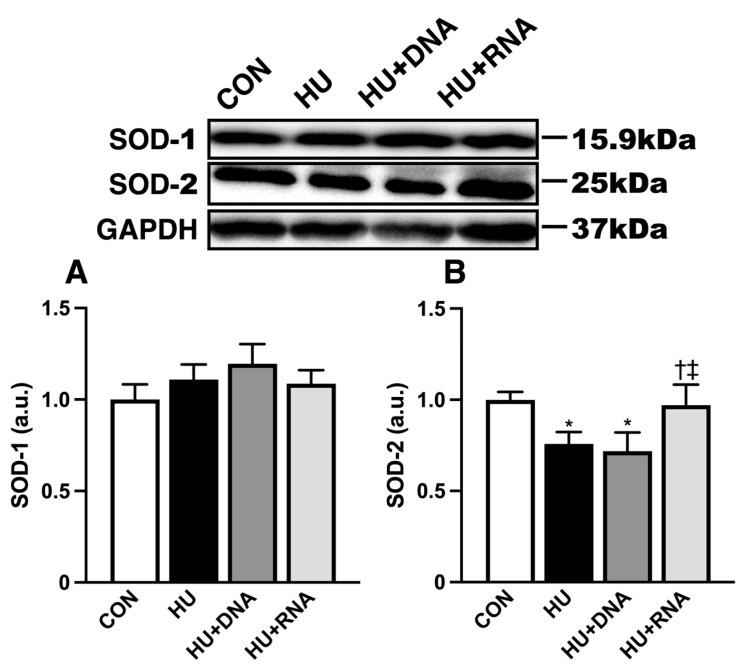
RNA treatment increases antioxidant enzyme protein SOD-2 expression in rats with hindlimb-unloading-induced atrophy. (**A**) Western blot result of SOD-1 expression and comparison of SOD-1 protein expression between the group. (**B**) Western blot result of SOD-2 expression and comparison of SOD-2 protein expression between the group. Values were calculated as fold changes relative to the control group. Values are expressed as mean ± SEM (n = 6). The symbols *, †, and ‡ indicate significant differences with respect to the CON, HU, and HU + DNA groups, respectively, at *p* < 0.05.

To evaluate oxidative stress, the SOD-1 and SOD-2 protein levels in the soleus muscle were quantified through SDS-PAGE and Western blot analysis. There was no discernible change (HU, HU + DNA, HU + RNA groups) in the expression levels of SOD-1 protein compared to the CON group (*p* = 0.8150, *p* = 0.4111, and *p* = 0.8937, respectively) (Figure 7A). The SOD-2 protein expression levels were significantly decreased following 14 days of HU (*p* < 0.05 vs. CON group), while the quantitative expression level in the HU + RNA group but not in the HU + DNA group remained consistent with the CON level (*p* < 0.05 *p* = 0.9267 vs. HU group, respectively) (Figure 7B).

## 4. Discussion

The innovative findings of this study reveal the following: (1) hindlimb unloading, characterized by chronic reduced loading and activity levels, resulted in increased oxidative stress (indicated by decreased SOD-2 levels) and elevated expression of anti-angiogenic regulatory factors (exemplified by TSP-1), resulting in capillary regression within the soleus muscle; (2) a medium concentration of RNA supplement may exert a more pronounced effect compared to DNA supplementation in mitigating capillary regression through the suppression of oxidative stress during chronic unloading.

During the 2-week unloading phase, the soleus muscle experienced atrophy along with capillary regression, as evidenced by a decline in fiber cross-sectional area and capillary-to-fiber ratio, respectively. It is well established in the literature that muscle mass and size decrease during periods of inactivity, resulting in muscle atrophy [25]. Moreover, atrophied muscles exhibit a decline in capillary number along with an elevation in oxidative stress, attributed to the excessive production of ROS [26]. Consequently, an oxidative stress-induced rise in ROS levels leads to the oxidation of membrane phospholipids, proteins, and DNA [27], facilitating vascular dysfunction [28]. The ROS generation rates critically influence the response, with moderate and optimal ROS levels being necessary for post-ischemic neovascularization, and excessive ROS hampering neovascularization [29]. Oxidative stress induces the upregulation of the TSP-1 protein [30], an anti-angiogenic factor. Research has shown that the enhanced expression of the TSP-1 protein triggers a restriction in endothelial cell growth [31] and neovascularization in vitro and in vivo [32,33]. Indeed, increased TSP-1 protein expression has been observed within the soleus muscle under unloading conditions [34]. Thus, the observed capillary regression in the HU group might be related to TSP-1 upregulation resulting from heightened oxidative stress, in the present study.

Although DNA has been recognized for its potential antioxidant capacity, it is noteworthy that the medium concentration of DNA, in our study, did not exhibit the ability to prevent the observed raise in ROS levels and the concurrent reduction in SOD-2 protein expression. In our previous investigation, we found that combining nucleoprotein, including DNA, with intermittent loading was effective in preventing capillary regression under conditions of chronic unloading. However, the exclusive supplementation of nucleoprotein, including DNA, under unloading conditions was inadequate to counteract capillary deterioration or attenuate the increase in oxidative stress [8]. The medium concentration of DNA resulted in an increased SDH activity, reflecting enhanced mitochondrial oxidative capability [35]. Building upon this, Marcu et al. presented compelling evidence supporting the role of mitochondrial function in modulating the ability of endothelial cells to promote angiogenesis, with specific emphasis placed on investigating the significance of mitochondrial proteins directly involved in the angiogenesis process [36]. However, the decrease in the TSP-1 level and the preventive effect of capillary regression were not observed in the HU + DNA group. Thus, we speculate that the medium concentration of DNA may be insufficient to prevent capillary regression, since the slow fiber composition in the HU + DNA group was not changed.

The antioxidant effects of monoribonucleotide-rich yeast hydrolysates were examined using the *Caenorhabditis elegans* nematode model [37]. RNA consists of AMP, CMP, GMP, and UMP, while UMP is not present in DNA. Notably, UMP has been identified as the most potent component for suppressing inflammation and enhancing mitochondrial function [38]. To assess the antioxidative effects of DNA and RNA supplementation, SOD-like activity measurement using WST-1 was performed. The results of our study indicated that RNA induced stronger SOD-like activity compared to DNA at concentrations of 0.5%, 1%, and 2%. Capillary regression associated with hindlimb unloading is modulated by a dysregulation in the equilibrium of pro- and antiangiogenic signaling pathways, characterized by the downregulation of VEGF and the upregulation of thrombospondin-1 [4]. In addition, treatment with the medium concentration of RNA attenuated the increase in the ROS (reflected in the intensity of DHE fluorescence and the expression levels of SOD-2 protein) and TSP-1 levels. These observations indicate that RNA confers defense effects against oxidative stress induced by unloading within the muscle. We also observed that RNA attenuated the impact of unloading on angiogenesis-suppressing factors and capillary density within the skeletal muscle, while untreated HU rats exhibited a reduction in capillary volume. However, the expression level of the angiogenic factor VEGF showed no significant differences among the CON, HU, HU + DNA, and HU + RNA groups, which aligns with our previous findings after 2 weeks of hindlimb unloading [34]. In addition, Olfert demonstrated that capillary regression is contingent upon elevated TSP-1 expression, rather than a reduction in VEGF expression [13]. The current findings indicated that the RNA treatment decreased the TSP-1 content and consequently attenuated the regression of capillaries in the examined skeletal muscle.

Angio-adaptation is strongly correlated with oxidative metabolism, muscle fiber type distribution, and the expression profile of oxidative stress [39,40]. And the muscle fiber type profile in the soleus muscle shows a positively correlated pattern with the capillary-to-fiber (C/F) ratio, indicating an association between fiber composition and capillarization [41]. In the present study, the proportion of slow-twitch fibers in both HU and HU + DNA groups was reduced, aligning with the observed capillary regression, whereas both the slow-twitch fiber composition and the C/F ratio in the HU + RNA group were kept at the control level. Therefore, the treatment with RNA also stimulated the transformation of muscle fibers from glycolic fibers to oxidative fibers by enhancing mitochondrial oxidative enzyme activity in the muscle fibers prior to the C/F ratio increase.

One constraint of the present study is that we restricted our analysis of RNA antioxidant effects to oxidative enzyme activity and reactive oxygen species (ROS) levels. Previous research indicated that the glycerophospholipid metabolism pathway exerts a critical influence on the antioxidant capacity of RNA [37], while our primary interest was in comparing the effects of DNA and RNA on capillary regression. Further investigation is needed to explore the pathways through which RNA exerts its antioxidant properties.

In conclusion, the antioxidative capacity of RNA was demonstrated in skeletal muscle subjected to prolonged periods of diminished mechanical loading and reduced activity levels. The RNA treatment had a more pronounced effect compared to DNA supplementation in counteracting capillary regression resulting from muscle atrophy. This study highlights the significance of nutritional interventions during periods of disuse and may offer valuable insights into the strategic use of bioactive compounds derived from yeast.

## Figures and Tables

**Table 1:** Composition of the experimental diets.

RNA (Torula Yeast RNA)					g/100 g
component	AMP	UMP	GMP	CMP	Total
**Monoribonucleotides + oligoribonucleotides**	18.96	16.11	25.78	14.65	75.50
DNA (Salmon Milt DNA)					g/100 g
component	dAMP	dTMP	dGMP	dCMP	Total
**Monodeoxyribonucleotides + oligodeoxyribonucleotides**	20.02	22.60	20.24	15.00	77.86

Abbreviations: AMP, Adenosine 5′-monophosphate; UMP, Uridine 5′-monophosphate; GMP, Guanosine 5′-monophosphate; CMP, Cytidine 5′-monophosphate; TMP, Thymidine 5′-monophosphate.

**Table 2:** Bodyweight, soleus weight, and soleus muscle FCSA.

	Body Weight (g)	Soleus Muscle Weight (mg)	Soleus Muscle FCSA (μm^2^)
CON	184 ± 11.6	80.5 ± 4	2385.1 ± 45.1
HU	175 ± 3.6	46.5 ± 1.3 *	1010.1 ± 12.2 *
HU + DNA	176.7 ± 3.7	48.1 ± 1.5 *	1079.9 ± 13.4 *
HU + RNA	179.2 ± 3.6	48.7 ± 1.4 *	1087.4 ± 6.9 *

Values are means ± SEM (n = 6). Abbreviations: CON, control; HU, hindlimb-unloaded; HU + DNA, hindlimb-unloaded plus nucleotide; HU+RNA, hindlimb-unloaded plus ribonucleotide. The symbols * indicate significant differences with respect to the CON, at *p* < 0.05. Unloading (HU, HU + DNA and HU + RNA) resulted in a decrease in the soleus muscle weight and soleus muscle FCSA.

## Data Availability

The data that substantiate this study’s conclusions are obtainable from the corresponding author upon reasonable request.
